# Mechanism Suggests How HIV Protein Disrupts Immune Cell Migration

**DOI:** 10.1371/journal.pbio.0020023

**Published:** 2004-01-20

One of the cornerstones of immune system function is movement. When word spreads that a virus has entered the body, chemical signals tell lymphocytes to proliferate and travel to the site of infection. Efforts to combat HIV have focused on understanding how the virus disrupts this immune response in the hopes of developing drugs to block its replication as well as vaccines to control the virus itself. Toward this end, scientists are investigating how each of the virus's nine genes—which all appear to have multiple functions—contribute to HIV infection.

When HIV infects a cell, viral enzymes copy its RNA genes into DNA, which can then invade the infected cell's chromosomes. The viral DNA might lay dormant or it might use the cell to reproduce more viruses, which go on to infect other cells. The course of infection is determined by interactions between circulating T cells and antigen-presenting cells (cells that present evidence of infection), like macrophages, which may unwittingly aid the virus by transferring it to the T cells. Macrophages, for example, produce proteins that tell T cells to come check out an infection.

A viral protein called Nef sparked intensive research after observations that patients with a rare strain of HIV lacking Nef took a very long time to develop AIDS symptoms. Nef has been linked to molecules involved in macrophage- and other antigen-signaling pathways and may use the molecules to appropriate these pathways for its own ends—enhancing virulence by facilitating viral replication. How Nef does this is not entirely clear. Now Jacek Skowronski and his colleagues at Cold Spring Harbor Laboratory in New York have identified the key molecules that Nef enlists to coopt the signaling machinery of immune cells.

To understand how this might happen, biochemically speaking, Skowronski's lab first needed to determine which molecules Nef associates with. An adaptor protein, Nef does not directly catalyze reactions, but binds to enzymes that do. The researchers identified two proteins, DOCK2 and ELMO1, that form a complex with Nef. DOCK2 regulates enzymes, called Rac1 and Rac2, that are required for normal lymphocyte migration and antigen-specific responses. ELMO1 has also been shown to help DOCK2 activate Rac. Because DOCK2 activates Rac as part of two different signaling pathways—one activated by the T cell receptor, which mediates T cell activation, and one by a chemokine receptor, which controls T cell migration—the researchers investigated whether Nef could affect these important pathways by modulating Rac activity. They found that Nef in fact activates Rac by binding to the DOCK2–ELMO1 complex. And they went on to show that HIV uses these components of the chemokine receptor pathway to disrupt T cell migration. To generate an effective immune response, it is crucial that T cells travel to sites within lymphatic tissues where they interact with other lymphocytes. By inhibiting T cell migration, the researchers propose, Nef prevents these critical interactions, thereby providing a mechanism for stifling the immune response.

These results, the authors argue, provide the biochemical evidence that Nef targets a protein “switch” that can interfere with important aspects of T cell function. In this way, Nef subverts the immune response pathways controlled by receptors on the surface of T cells to effectively disarm the immune system and turn T cells into viral replication factories. Understanding how Nef interacts with these proteins to spread infection could lay the foundation for valuable new therapies aimed at inhibiting and arresting HIV infection by blocking Nef-mediated effects.

**Figure:**
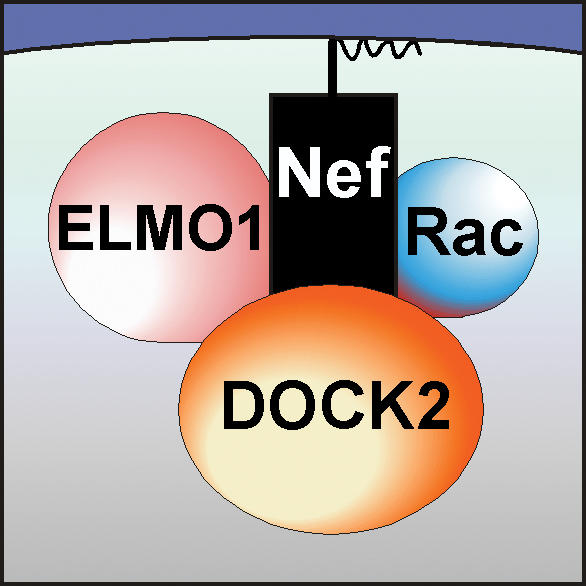
Pathway by which Nef disrupts T cell migration

